# Research progress on the depth of anesthesia monitoring based on the electroencephalogram

**DOI:** 10.1002/ibra.12186

**Published:** 2024-12-06

**Authors:** Xiaolan He, Tingting Li, Xiao Wang

**Affiliations:** ^1^ Department of Anesthesiology, West China Hospital Sichuan University Chengdu China

**Keywords:** consciousness, deep learning structure, electroencephalogram, the depth of anesthesia monitoring

## Abstract

General anesthesia typically involves three key components: amnesia, analgesia, and immobilization. Monitoring the depth of anesthesia (DOA) during surgery is crucial for personalizing anesthesia regimens and ensuring precise drug delivery. Since general anesthetics act primarily on the brain, this organ becomes the target for monitoring DOA. Electroencephalogram (EEG) can record the electrical activity generated by various brain tissues, enabling anesthesiologists to monitor the DOA from real‐time changes in a patient's brain activity during surgery. This monitoring helps to optimize anesthesia medication, prevent intraoperative awareness, and reduce the incidence of cardiovascular and other adverse events, contributing to anesthesia safety. Different anesthetic drugs exert different effects on the EEG characteristics, which have been extensively studied in commonly used anesthetic drugs. However, due to the limited understanding of the biological basis of consciousness and the mechanisms of anesthetic drugs acting on the brain, combined with the effects of various factors on existing EEG monitors, DOA cannot be accurately expressed via EEG. The lack of patient reactivity during general anesthesia does not necessarily indicate unconsciousness, highlighting the importance of distinguishing the mechanisms of consciousness and conscious connectivity when monitoring perioperative anesthesia depth. Although EEG is an important means of monitoring DOA, continuous optimization is necessary to extract characteristic information from EEG to monitor DOA, and EEG monitoring technology based on artificial intelligence analysis is an emerging research direction.

## Graphical abstract

Electroencephalogram (EEG) can noninvasive, continuous, and real‐time monitor the state of brain electrical activity, and the monitoring of EEG can reflect changes in the depth of anesthesia (DOA). The development of artificial intelligence can enable anesthesiologists to extract, analyze, and quantify DOA from complex EEG data. The future development of medical systems cannot be separated from the assistance of artificial intelligence.

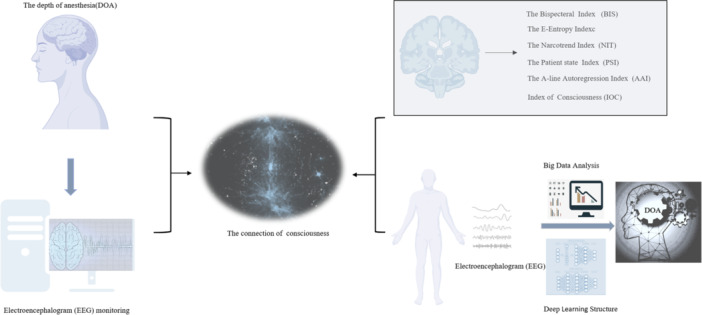

## 1 INTRODUCTION

In clinical practice, anesthesiologists regulate and maintain a certain depth of anesthesia (DOA) to ensure anesthesia safety by monitoring key signs, supporting the cardiovascular system, and monitoring organ perfusion and fluid flow. However, these processes are affected by several confounding factors, individual differences, and the subjective judgment of anesthesiologists. Given that managing the DOA during surgery is essential to ensure patient safety and optimal outcomes, researchers are actively developing methods to objectively and visually assess DOA. Since the brain is the primary target of anesthetic drugs, it has become a critical area for DOA monitoring. Electroencephalogram (EEG) holds promise as a real‐time monitor of brain activity, potentially reflecting changes in DOA. Nevertheless, due to brain complexity, the uncertainty of anesthesia mechanisms, and the specificity and sensitivity of EEG, EEG monitoring adoption remains in the infancy stages. The advent of artificial intelligence (AI) has revolutionized EEG monitoring by enhancing the ability to process large, complex data sets, enabling a shift from simple EEG indices to advanced AI analysis techniques. This review explores recent research on DOA, its association with EEG monitoring, and the integration of AI analysis with EEG for improved DOA assessment.

## 2 THE DOA AND THE DOA MONITORING

### 2.1 Definition and significance of the DOA

The term “depth of anesthesia” originated from ether anesthesia and refers to the depth of etherization.^1^ According to the leading anesthesia textbook, *Miller's Anesthesiology*, general anesthesia is a reversible drug‐induced state characterized by four key components: antinociception, unconsciousness, amnesia, and muscle relaxation. Because muscle relaxants are often used during general anesthesia, it becomes impossible to rely solely on a patient's physical movements in response to surgical stimulation to assess the adequacy of DOA. Therefore, independent monitoring of these components is crucial. Although the spontaneous behavioral responsiveness is lost in the subcortical area during light anesthesia, the corticothalamic network continues to generate consciousness through information integration and preserves connectivity without interfering with norepinephrine signal transmission^2^; at the same time, the patient may have the possibility of intraoperative awareness. Therefore, continuous monitoring of the DOA during anesthesia can guide the application of anesthesia drugs, maintain an appropriate level of consciousness with the lowest dose of anesthesia drugs, and prevent potential dangers caused by insufficient or excessive anesthesia drugs (intraoperative awareness, excessive suppression of the circulatory system, delayed postoperative recovery, etc.). Additionally, the real‐time changes in anesthesia drugs and surgical stimuli during surgery cause dynamic changes in the DOA. Therefore, it is necessary to continuously monitor and adjust this dynamic change during the perioperative period.

### 2.2 The DOA monitoring

General anesthesia aims to satisfy the patient's surgical experience and prevent the harmful stimulation caused by surgery from arousing the central nervous system and causing cardiovascular and neurohumoral system reactions. Considering that amnesia, analgesia, and immobilization are elements of general anesthesia, reasonable monitoring of DOA should also revolve around these key elements. The monitoring techniques and guidelines for neuromuscular function have become relatively mature and broadly applied in clinical practice.^3^ However, the monitoring of amnesia and analgesia remains under investigation and improvement.

**Figure 1:**
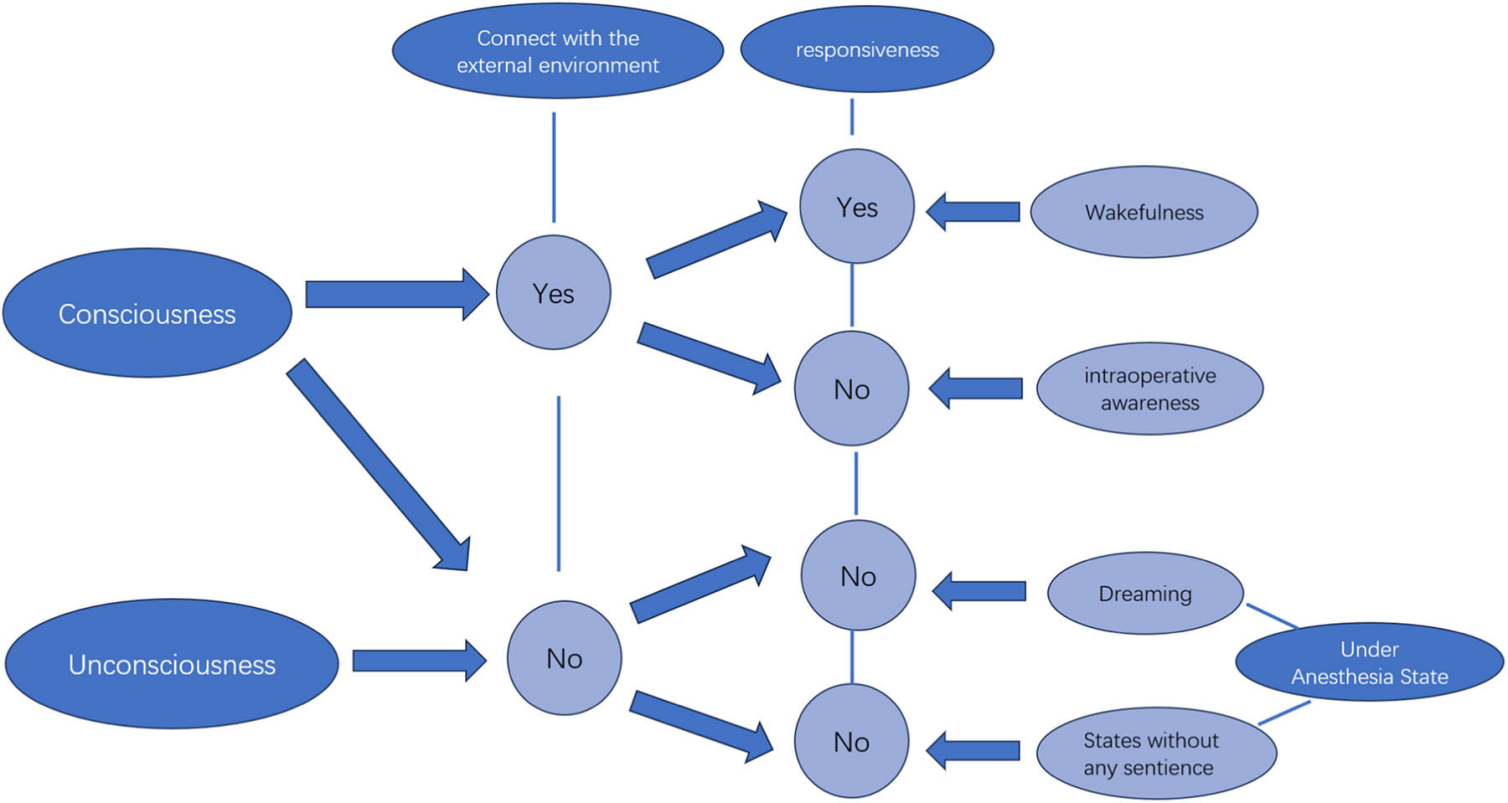
The states of connected consciousness, disconnected consciousness, and unconsciousness. Unresponsiveness does not necessarily mean unconsciousness, and patients who are unresponsive during general anesthesia may experience intraoperative awareness. During general anesthesia, patients may dream, but it is not related to the depth of anesthesia.^2^, ^4^, ^5^ [Color figure can be viewed at wileyonlinelibrary.com]

During surgery, general anesthesia induces a state of unconsciousness, effectively separating a patient's awareness from the environment to prevent them from recalling the experience. Understanding the exact changes in consciousness during general anesthesia is critical for anesthesia management (Figure 1).^2^ The full role that consciousness plays within the brain is still under debate. Multiple theories have been proposed to explain consciousness, each focusing on distinct characteristics. Some of these prominent theories, like the Global Neuronal Workspace Hypothesis^6^ and the Integrated Information Theory,^7^ are not without their critics. The reversible changes in consciousness triggered by anesthetic drugs offer a valuable tool for investigating the nature of brain consciousness. By analyzing the characteristic alterations in the brain's functional state caused by these drugs, researchers can potentially develop methods to monitor the conscious state during anesthesia. Studies have demonstrated that the thalamus, cingulate cortices, and angular gyri are essential to human consciousness.^4^ The activity of the central lateral thalamus and cortical deep layers modulate consciousness. As a previous study on macaques revealed, reactivation of central lateral thalamus‐deep cortical circuitry by gamma frequency stimulation during general anesthesia can restore awakening‐like cortical dynamics and improve consciousness level.^8^ Another animal study also pointed out that during the onset and recovery from anesthesia (three anesthesia schemes: isoflurane [Iso], Fentanyl‐Medetomidine‐Midazolam, and Ketamine‐Xylazine‐Iso), the changes in the deep cortex are consistent with the loss and recovery of consciousness.^9^ Anesthetics may alter the feedback loop between the thalamus cortex and the cortex by affecting the fifth layer of cortical pyramidal neurons, causing a loss of consciousness. This mechanism may represent a common target for different types of general anesthesia drugs, while changes observed in other cortical layers and inhibitory neurons may be drug‐specific.^10^ It is also worth noting that patients who do not respond during general anesthesia may still retain consciousness, potentially experiencing external environmental stimuli (intraoperative awareness) or an internal stimulus experience (dream). However, in most cases, dreaming during anesthesia is harmless, enjoyable, and unrelated to the DOA.^11^ Therefore, satisfactory general anesthesia can be achieved through loss of consciousness or disconnection of consciousness connectivity. Differentiating consciousness and consciousness connectivity mechanisms can help monitor general anesthesia and formulate anesthesia plans.

The International Association for the Study of Pain defines “pain as an unpleasant sensory and emotional experience associated with actual or potential tissue damage.”^12^ This definition indicates that pain not only includes the subjective feelings of patients in a conscious state but also the objective response of the body to nociceptive stimuli. Monitoring of nociceptive stimuli can help optimize perioperative pain management by reducing the occurrence of intraoperative stress reactions, minimizing the use of opioid drugs, and identifying high‐risk individuals for postoperative pain. Body response to nociceptive stimuli can be manifested in various ways, including physical response (spinal cord reflex that causes limb movements), autonomic reflex (brain stem and hypothalamus action causing changes in heart rate, ventilation frequency, vasoconstriction, and pupil), cognitive arousal, and memory formation (the subcortical and midbrain structures cause overall arousal, mediating attention and memory formation through the forebrain and cortex), various endocrine, coagulation, and immune‐inflammatory reactions.^13^ Therefore, nociceptive stimuli monitoring can be divided into three categories based on the following: motor reflexes, the central nervous system, and the autonomic nervous system. Currently, few studies have investigated methods for improving pain monitoring during general anesthesia, and current technologies and indices that predict responses to nociceptive stimuli are limited by several factors. For example, the Analgesia Nociception Index (qNOX) relies on heart rate variability (HRV), which reflects parasympathetic activity. However, its reliability is compromised when drugs like atropine and ephedrine, which affect HRV, are administered.^14^ The skin conductance is influenced by central and peripheral modulators of the autonomic nervous system including neuromuscular reversal agents and alpha‐2‐agonists,^15^ while measurement of the pupillary diameter is affected by miotic effects of opioids.^16^ Additionally, indices such as the qNOX based on electromyography can be influenced by muscle relaxants, further complicating accurate monitoring.^17^ Moreover, the nociceptive flexion reflex, a polysynaptic spinal withdrawal reflex elicited by the activation of nociceptive A‐delta afferents, is also limited by the complexity of its setup and complicated procedures.^18^ Thus, the monitoring of nociceptive stimuli still requires continuous and additional research.

Although perioperative unconsciousness and analgesics may exert a synergistic effect, nociceptive stimuli due to insufficient analgesia can overwhelm the effects of sedative and hypnotic drugs, thereby “awakening” patients. Distinguishing the need for sedation or analgesia during general anesthesia is crucial for managing DOA. Notably, changes in consciousness and pain can alter EEG activity, making EEG‐based monitoring a valuable tool. This dual‐purpose monitoring can simultaneously track both aspects, offering comparable data. The American Society for Accelerated Rehabilitation and the Perioperative Quality believes that EEG monitoring in perioperative anesthesia management should be considered a significant organ monitoring.^19^ EEG monitoring is noninvasive and continuous, which can simultaneously monitor analgesic and sedative components while reducing additional consumption costs and associated risk. Studies have shown that EEG changes induced by nociceptive stimuli (*β* arousal, *δ* arousal, *α* dropout) reveal the DOA, indicating the need for enhanced pain relief, including opioid administration or local anesthesia blockade, rather than an increase in sedative use.^13^ At present, these studies still lack adequate evidence, and therefore, EEG monitoring during anesthesia warrants further investigations.

## 3 EEG AND ANESTHESIA‐RELATED EEG‐DERIVED INDICES

### 3.1 EEG

EEG records change in voltage potentials generated by electrical currents in and around brain neurons via electrodes placed on the scalp.^20^ It was first discovered by the German neurologist and psychiatrist Hans Berger in 1924 and published in 1929.^21^ At present, EEG is one of the standard methods for measuring brain activity in many fields, with a degree of continuity and noninvasiveness, similar to the response of electrocardiogram to the electrical activity of the heart, by integrating the potentiometric activity in the cortex and subcortical structures of the brain.

The human brain contains about 100 billion neurons, each with about 10,000 connections to other neurons, forming a huge, electrically active neural network.^22^ Superposition and synchronization of electrical activity between neurons generate local field potentials (LFP). However, when these LFPs reach the scalp, they are attenuated by the weakening of the electric field with the square of the distance and the spatial filtering effects of head tissues (brain, cerebrospinal fluid, skull, scalp).^23^ Thus, EEG is an artificially modified recording of LFP. Notably, EEG does not measure the action potentials, but relatively slow postsynaptic potentials generated by the release of neurotransmitters at the axon neural terminals.^20^ Because of neurotransmitters, synaptic potentials can be classified as excitatory or inhibitory, and the EEG is the sum of the net effect of excitatory postsynaptic potentials or inhibitory postsynaptic potentials during the synchronization of many neurons recorded through scalp electrodes.^24^

### 3.2 EEG waveforms and EEG artifacts

**Figure 2:**
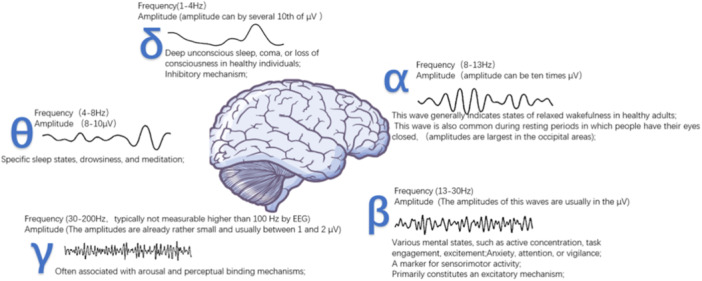
Common electroencephalogram (EEG) waveforms and their clinical significance. Based on frequency and power, the EEG is categorized into distinct frequency bands, that is, *δ*, *β*, *α*, *θ*, and *γ*. Different waveforms represent different clinical meanings.^22^ [Color figure can be viewed at wileyonlinelibrary.com]

The EEG depicts neural electrical activity in the brain with time‐lapse as the horizontal axis and potential changes as the vertical axis, containing basic elements including frequency, amplitude, and phase. Based on frequency and power, it is categorized into distinct frequency bands, that is, *δ*, *β*, *α*, *θ*, and *γ* (Figure 2). EEG can be influenced by age, state of consciousness, mental activity, drug effects, and brain diseases, making it a tool in disease diagnosis through proper interpretation.^8^, ^9^ Despite its high diagnostic value and specificity, EEG is highly susceptible to external factors, which can introduce artifacts into the recorded signals.^25^ These artifacts, which are not generated by brain activity, can be categorized as physiologic and extra‐physiologic. Physiologic artifacts originate from body parts outside the brain, including electromyographic (EMG) artifacts, electrooculography artifacts, motion artifacts, electrocardiographic (ECG) artifacts, and sweat artifacts, while extra‐physiological artifacts arise from external sources and can be categorized into environmental factors (electrodes, devices, and cell phones) and in vivo devices factors (pacemakers and neurostimulators).^26^ Cell phones, for instance, can introduce electromagnetic interference into the EEG signal. On the other hand, powerful sources like television transmitters can overwhelm the amplifiers entirely, rendering the data unusable.^27^ With comprehensive research on EEG and the advancement of technology, it is currently possible to identify and distinguish some normal rhythms, artifacts, and a small number of normal patterns, thereby improving the accuracy of EEG analysis.

### 3.3 EEG studies of several common classes of anesthetic drugs

**Figure 3:**
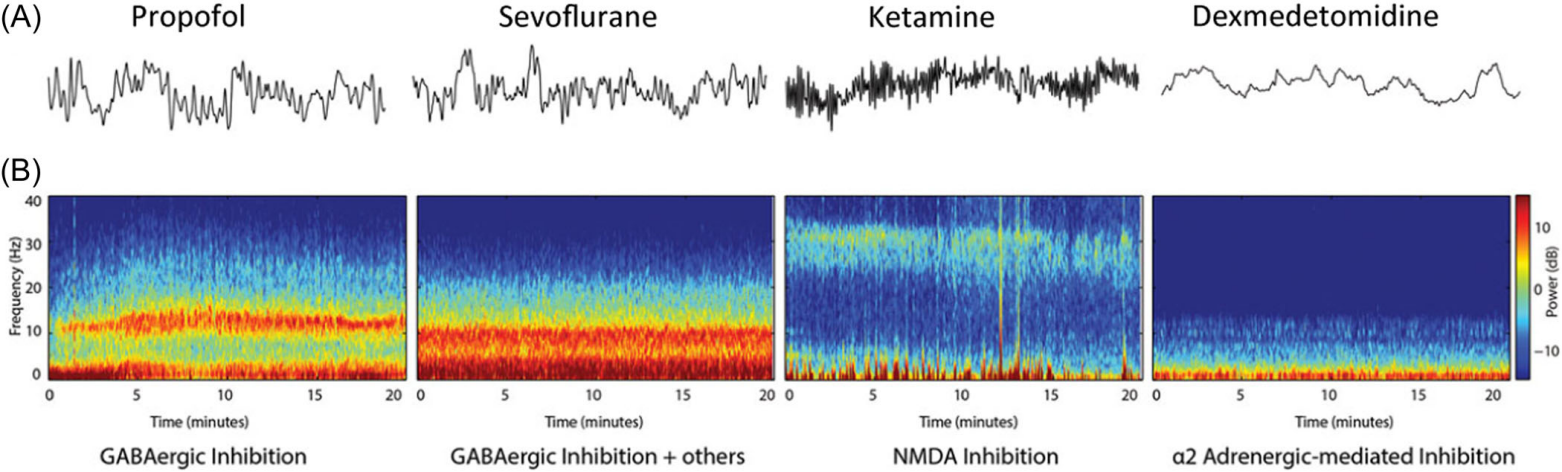
Electroencephalogram (EEG) traces (A) and spectrograms (B) characteristics of several commonly used anesthetics. The use of anesthetic drugs can cause significant changes in EEG oscillations. The EEG characteristics caused by several common types of anesthetic drugs have been studied. By using spectrograms, its features can be more clearly represented.^28^ Figure cited from Clinical Electroencephalography for Anesthesiologists Part I: Background and Basic Signatures. doi:10.1097/ALN.0000000000000841. [Color figure can be viewed at wileyonlinelibrary.com]

General anesthetics cause a reversible loss of consciousness by targeting several neural circuits in the cortex and subcortical regions. However, their precise functional profile remains to be fully understood. Different anesthetic drugs, owing to their distinct molecular mechanisms, elicit varying changes in EEG waveforms, each producing a unique EEG pattern. The EEG generated by several commonly used anesthetic drugs has been studied and well described (Figure 3).^28^ Propofol reacts with *γ*‐GABA_A_ receptor binding and enhances postsynaptic neuronal hyperpolarization, regularly occurring during sedation *β*–*γ* oscillation and slow *δ* oscillation, causing unconsciousness *δ* wave combined with *α* wave. Inhalation anesthetics like sevoflurane, Iso, and desflurane achieve their effects through various mechanisms, including the enhancement of GABAergic inhibition, blocking of the specific ion channels, and limiting glutamate release. As a result, these anesthetics produce distinct EEG patterns characterized by *α*, slow‐*δ*, and *θ* oscillations. Ketamine, an anesthetic with analgesic effects, binds to N‐Methyl‐d‐Aspartate receptors in the brain and spinal cord, increasing the metabolic rate of the brain, cerebral blood flow, and the production of hallucinations. At low doses, ketamine alone can induce faster EEG wave patterns, specifically *β*–*γ* oscillations ranging from 25 to 32 Hz. Dexmedetomidine activates *α*_2_ receptors to reduce the release of locus ceruleus norepinephrine to produce sedation, which produces characteristic slow delta and spindle waves.^28^

These described EEG characteristics correspond to the administration of a single anesthetic at a particular dose. However, general anesthesia often involves the use of multiple drugs in various combinations, which complicates the interpretation of EEG patterns. The EEG changes with the dose, type, and combination of anesthetics, making it improbable that a single EEG characteristic is universally applicable across all ages, drug combinations, and patient conditions. For instance, when ketamine is used alongside GABAergic anesthetics, brain waves in the *β* range are also activated in the context of the *α*/*δ* pattern. When dexmedetomidine is used in conjunction with GABAergic anesthetics, the *α*/*δ* pattern is mainly characterized by an increase in the *δ* wave. However, it is worth noting that, in the case of sufficient anesthetic concentration, insufficient analgesia also causes the disappearance of the *α*/*δ* wave, an increase in the high‐frequency waves (*β* and *γ*), and an increase in *δ*‐wave.^29^ These changes will lead to changes in the values of the monitoring instrument, potentially causing anesthesiologists to misjudge the DOA, which might result in patients being subjected to deeper levels of anesthesia or insufficient use of analgesics. Therefore, anesthesiologists should also assess the drug combinations and patients' specific status when using EEG to monitor brain function.

### 3.4 Anesthetic‐related EEG‐derived indices

Since interpreting raw EEG signals can be complex and time‐consuming for clinicians, researchers have developed a series of simplified scores. These scores, called dimensionless indices, are calculated by applying mathematical formulas to specific features within the raw EEG data. So far, more than 10 anesthesia‐related EEG monitoring devices have been well described and studied, each possessing distinct characteristics due to differences in how they analyze raw EEG data and the algorithms used to derive their respective indices (Table 1). The most popular EEG monitor is the commercially available Bispectral index (BIS), approved by the US Food and Drug Administration in 1996 for monitoring DOA. BIS combines time‐domain, frequency‐domain, and bispectral analyses of the EEG and is shown as a dimensionless number between 0 (deep anesthesia) and 100 (wakefulness), where 40–60 applies to surgical anesthesia.^36^ BIS monitoring has been shown to reduce the incidence of intraoperative awareness among patients undergoing general anesthesia.^30^ Meanwhile, some studies suggest that perioperative BIS monitoring also reduces the incidence of postoperative delirium as well as cognitive dysfunction.^37^ Although BIS monitoring offers some advantages, it has limitations and can be influenced by various factors, leading to concerns about its effectiveness in improving outcomes like delirium and cognitive dysfunction.^38^ First, BIS has a delay of about 30–60 s in real‐time monitoring of changes in the DOA.^39^ Second, BIS can be affected by the type of anesthetic drug, age, body temperature, neurological disorders, and other medical devices.^40^ For instance, while BIS is an effective measure of the depth of sedation when using propofol, etomidate, midazolam, and volatile inhalation anesthetics alone,^41^ it is ineffective for drugs such as ketamine, dexmedetomidine, and nitrous oxide (N_2_O).^36^, ^42^, ^43^ The BIS values have been confirmed for healthy adults, and there is uncertainty about the index for brains that are maturing and beginning to form synapses, as well as for aging brains.^44^, ^45^ One study also noted that the BIS decreases by 1.12 units for every degree Celsius decrease in body temperature during extracorporeal circulation.^46^

**Table 1:** Common EEG‐derived indices and clinical significance.

Common EEG‐derived indexes	Clinical characteristics
The BIS index^30^	**Dimensionless Number**: 0 (deep anesthesia)–100 (awake) Recommended target range during general anesthesia: 40–60
The E‐Entropy index^31^	**RE** (The frequency calculation range is 0.8–47 Hz; Combining EEG and EMG data to include cortical and frontal skeletal muscle activity) Index scale: 0–91; Recommended target range during general anesthesia:40–60 **SE** (The frequency calculation range is 0.8–32 Hz; Using only EEG data to specifically represent cortical activity and reflect hypnotic depth) Index scale: 0–91; Recommended target range during general anesthesia:40–60 **Δ (RE–SE)** > **10**: Patients may experience awakening
The NTI^32^	**Index scale**: A (awake)‐F (general anesthesia with increasing burst suppression); divided into 14 substages (A, B_0–2_, C_0–2_, D_0–2_, E_0,1_, F_0,1_); A (awake), B (sedated), C (light anesthesia), D (general anesthesia), E (general anesthesia with deep hypnosis), F (general anesthesia with burst suppression)
The PSI^33^	**Dimensionless number:** 0 (deeply anesthetized)–100 (fully awake) Recommended target range during general anesthesia:25–50
The AAI^34^	**Dimensionless number**: 0 (deep anesthesia)–100 (awake) 0–15 (deep anesthesia) 15–25 (surgical anesthesia with a peak at 20) 25–40 (light anesthesia, denoting intraoperative awareness) above 50 (awake level)
IOC^35^	**IoC**_**1**_ (The index of depth of sedation):0–99 40–60: Recommended target range during the operative period; above 60: indicating insufficient use of sedative agents; <40: excessive sedation **IoC**_**2**_ (The index of depth of analgesic):0–99 30–50: Recommended target range during the operative period; above 50: indicating insufficient use of analgesic agents, <30: indicating excessive analgesic effects

Abbreviations: AAI, A‐line autoregression index; BIS, bispectral; EEG, electroencephalogram; EMG, electromyographic; IOC, index of consciousness; NTI, Narcotrend index; PSI, patient state index; RE, response entropy; SE, state entropy.

The index of consciousness (IOC) is another method for monitoring DOA based on EEG analysis, and the main parameters are computed through symbolic dynamics methods.^47^ IOC is categorized into IoC_1_ (quantum consciousness index, qCON) and IoC_2_ (injury sensitivity index, qNOX). IoC_1_, which is used to assess the sedation status of patients, correlates well with BIS; IoC_2_, which is used to assess the depth of analgesia, is useful in guiding perioperative analgesic use.^35^, ^48^ The patient state index (PSI) is computed from four different electrodes and an additional reference electrode and ground electrode, which combines information from various brain regions to indicate global and regional brain states. The PSI algorithm was developed based on existing patient databases and quantitative EEG information from clinical cases. PSI‐guided induction, maintenance, and extubation of general anesthesia reduces the use of anesthetic medications and has less electrode interference during the procedure.^49^ Unlike BIS, the PSI algorithm has higher sensitivity and specificity for altered consciousness during induction and awakening of general anesthesia.^50^ Entropy is a mathematical concept for interpreting nonlinear dynamic data that combines two techniques EEG signal and facial EMG analysis. It is divided into two parts, state entropy (SE) and response entropy (RE).^51^ RE includes EEG and frontal electromyography, with electrical activity in the frequency range of 0.8–47 Hz, while SE contains irregular EEG activity in the frequency range of 0.8–32 Hz. RE can help researchers keep a balance between inadequate anesthesia and patient arousal during painful stimuli.^52^ However, entropy cannot be used in ketamine and N_2_O anesthesia management.^53^ The Narcotrend algorithm is derived from a system developed for visual typing of EEG patterns associated with natural sleep states, distinguishing from A (awake) to F (burst suppression to subcortical silencing). The latest version of the Narcotrend index displays a range from 0 (EEG silence) to 100 (awake).^54^ Auditory evoked potentials (AEPs) resemble tiny electrical echoes in the brain. These echoes are created when your brain responds to sounds, and they reveal how electrical activity travels along the auditory nerve pathway. AEPs typically respond to an increase in anesthesia concentration with increasing signal latency and decreasing signal amplitude. The mid‐latency AEP (MLAEPs) with a latency of 10–15 ms show graded changes with increasing anesthesia depth, revealing a certain correlation with anesthesia depth. The A‐line autoregression index of 0–100 can be derived by analyzing and computing signals of MLAEPs.^55^, ^56^ Research has shown that MLAPEs have a faster response speed than that of BIS.^57^

Despite evidence suggesting the benefits of EEG‐derived indices for monitoring DOA, they have not become the standard practice in clinical settings. First, one significant limitation is that the use of these indices does not fully prevent intraoperative awareness of general anesthesia. Second, these indices were developed on an adult basis, rendering them less reliable for use in the pediatric population. Third, these indices do not directly correlate with the neurophysiology mechanisms by which specific anesthetics act on the brain, leading to an imprecise depiction of the brain's response to these drugs. Finally, these indices are based on changes in EEG oscillations caused by high‐dose intravenous or inhaled anesthetics, in which, slower oscillations represent deeper anesthesia states.^28^ However, currently, there are three types of anesthetic drugs that have raised doubts about these indices. Ketamine and NO_2_ produce faster oscillations under deeper anesthesia; dexmedetomidine produces slower oscillations under deeper anesthesia.^42^, ^43^ It is important to note that current clinical tools for monitoring DOA (BIS, Entropy‐SE, Narcotrend, qCON) can sometimes produce conflicting recommendations even when analyzing the same brainwave patterns. This inconsistency underscores the challenge of achieving a uniform and accurate reflection of a patient's anesthetic state across different monitoring devices.^58^, ^59^ Therefore, the use of these EEG‐derived indices should be rationalized by considering their advantages and disadvantages in terms of different clinical settings.

## 4 FUTURE DEVELOPMENTS AND DIRECTIONS IN DOA MONITORING

**Figure 4:**
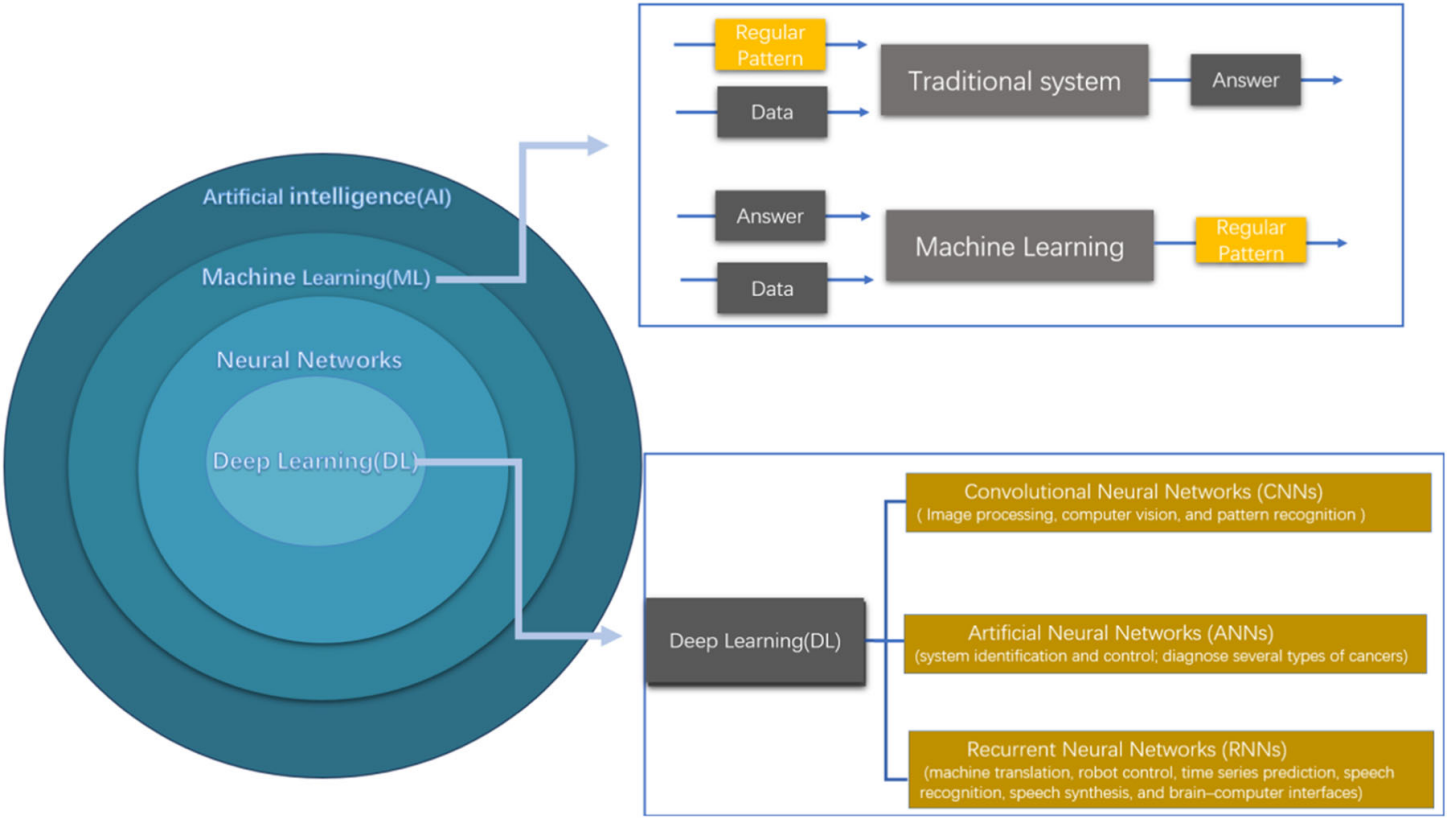
The relations between deep learning (DL), neural networks, machine learning (ML), and artificial intelligence (AI). An important branch of AI is ML. Different from traditional systems, it does not require explicit commands to draw conclusions. Instead, it automatically learns from data and improves from experience. DL is a subset of ML, which generates predictions from a data set using multilayer neural networks. DL includes convolutional neural networks (CNNs), artificial neural networks (ANNs), and recurrent neural networks (RNNs). Due to their different characteristics, they are applied in various clinical fields.^65^ [Color figure can be viewed at wileyonlinelibrary.com]

We previously discussed the importance of monitoring DOA to achieve optimal general anesthesia, which requires amnesia, analgesia, and immobilization. Since a single index might not capture all aspects of DOA effectively, it is crucial to explore separate indices that reflect each of these essential components.^60^ Although anesthesiologists can benefit from raw EEG, the complexity of this series makes it difficult to obtain information from EEG due to varying patient conditions under general anesthesia as well as the combination of anesthesia drugs, and the nonlinear relationship between the DOA and raw EEG. Many studies have also ascertained that current EEG monitoring devices have limitations, and AI has become an important research tool for EEG analysis or DOA monitoring due to its powerful data processing and self‐learning ability. Machine learning (ML) is an important branch of AI that allows computers to learn from data without needing explicit instructions. This data can come in many forms, including numbers, images, text, and even sounds. Neural networks, inspired by the structure of the brain, are a popular type of ML technique.^61^ Each network is made up of an input layer (to describe the data), at least one hidden layer (to conduct different mathematical transformations on the input features), and an output layer (to yield a result).^62^ When the neural network contains more than one hidden layer, it is called a deep neural network. Deep neural networks can reuse the features computed in a given hidden layer in higher hidden layers. Deep learning (DL) refers to the process of automatically determining the parameters deep within a network based on experience (data). This allows the network to develop self‐learning features based on the data itself.^63^, ^64^ DL includes convolutional neural networks (CNNs), artificial neural networks, and recurrent neural networks.^65^ Currently, many DL structures have been applied to EEG analysis (Figure 4).^66^

Several studies in DOA monitoring have shown that the accuracy and speed of DOA assessment by deep‐learning neural networks are superior to conventional methods.^67^ The DL model proposed by Shi M et al. directly extracts 14 features from EEG signals as inputs and uses PSI as reference output to estimate DOA. Its superiority and prospects have been confirmed by comparing it with three traditional models.^68^ The DL structural model proposed by Sara et al., which involves a CNN combination, has a data set containing patients with various features and different types of anesthesia. This model continuously predicts a patient's BIS using EEG signals. It achieves an impressive accuracy of 88.7% in distinguishing four anesthesia states: awake, mild anesthesia, general anesthesia, and deep anesthesia, surpassing the performance of conventional methods.^69^ The EEG‐based minimum alveolar concentration is a novel metric designed by Yongjae et al. for DOA assessment in patients with inhalation anesthetics and intravenous anesthetics, which is based on the minimum effective alveolar concentration of EEG. The model comprises four‐layer CNN and two dense layers as a deep‐learning protocol for simultaneous administration of both anesthetics, and the metric is faster compared to the latency of BIS.^70^ Ahmad Shalbaf et al proposed a novel automated method for assessing the DOA. This method selected the best subset from 11 features filtered from EEG signals. This subset was used as input for the Adaptive Neuro‐Fuzzy Inference System with Linguistic Hedges (ANFIS‐LH), and ANFIS‐LH is a new neuro‐fuzzy classification algorithm. Compared with the RE index, its accuracy in distinguishing four anesthesia states is 92%.^71^ Several studies have confirmed that AI offers significant advantages over traditional methods, particularly when dealing with complex and nonlinear data sets like EEG. AI can rapidly uncover hidden correlations within the data that might escape human interpretation. However, AI development faces challenges. First, deep neural networks require vast amounts of data for training and testing. This data collection and application process can raise concerns about data leakage and potential exposure to patient privacy.^62^, ^63^ Second, when testing these data sets, an accurate definition or standard should be adopted as this will determine the accuracy of AI output. When the standard itself is questioned, the results obtained by AI may be incorrect. For instance, as discussed earlier, concerns exist regarding the EEG index itself. This raises a question: could the AI model designed using this index as a DOA evaluation standard also be problematic?^72^ Finally, while “transparent” learning methods allow users to assess the accuracy of AI results, some models remain “black boxes.” These models cannot provide clear explanations for the reasoning behind their outputs. This lack of transparency is particularly concerning in the field of anesthesia, where even minor errors can have severe consequences.^73^ In summary, the combination of DOA monitoring and AI is an inevitable trend in the future. Its superiority has been gradually appreciated despite currently being in the exploratory stage. For effective AI applications in DOA monitoring, two factors are crucial. First, the model design and data analysis must be accurate. Second, we need a standardized definition of DOA and a clear way to classify consciousness levels.

## 5 CONCLUSION AND OUTLOOK

Although existing research has not completely uncovered the mystery of anesthesia mechanisms, understanding these mechanisms is essential for the effective monitoring of anesthesia depth. Investigating anesthesia mechanisms provides valuable insights that can guide the development of more accurate monitoring techniques, particularly through the study of EEG. EEG, which monitors brain electrical activity much like ECG monitors for heart electrical activity, is a critical tool in this research. However, the brain's complexity far exceeds that of the heart, necessitating further exploration of the relationship between EEG patterns and brain function, as well as methods to extract clinically useful information from EEG data. With the current development of computers and AI in healthcare, we can rely on their powerful data processing and self‐learning abilities in the future to automatically extract appropriate EEG signals from the original EEG based on patient status, medication combination, anesthesia methods, and surgical methods. AI has the potential to improve perioperative management by analyzing the surgical environment and selecting more appropriate monitoring plans. However, we note that AI serves as an auxiliary diagnostic tool. It cannot replace the expertise of clinical physicians in the entire medical field, including DOA monitoring. Clinical decisions are often made based on the physician's knowledge and experience, especially when dealing with complex or rare cases in clinical practice. Therefore, when designing AI models, developers need to establish clear guidance standards. Similarly, users must understand the model's core operating principles to critically evaluate the accuracy of its results. AI technology is rapidly evolving, showcasing its vast potential. Future advancements lie in creating even larger databases to enhance the self‐learning capabilities of these models. Additionally, standardized reference data for related models is crucial to ensure their accuracy.

## AUTHOR CONTRIBUTIONS

Xiaolan He is responsible for data curation and original draft writing. Tingting Li and Xiao Wang have participated in writing, reviewing, and editing. All authors read and approved the final manuscript.

## CONFLICT OF INTEREST STATEMENT

The authors declare no conflict of interest.

## ETHICS STATEMENT

Not applicable.

## Data Availability

Data sharing is not applicable to this article as no new data were created or analyzed in this study.
